# Preparation, Stabilization and Carbonization of a Novel Polyacrylonitrile-Based Carbon Fiber Precursor

**DOI:** 10.3390/polym11071150

**Published:** 2019-07-04

**Authors:** Huichao Liu, Shuo Zhang, Jinglong Yang, Muwei Ji, Jiali Yu, Mingliang Wang, Xiaoyan Chai, Bo Yang, Caizhen Zhu, Jian Xu

**Affiliations:** Institute of Low-dimensional Materials Genome Initiative, College of Chemistry and Environmental Engineering, Shenzhen University, Shenzhen 518060, China

**Keywords:** carbon fiber, PAN-based precursor, 2-acrtlamido-2-methylpropane acid, thermal oxidative stabilization

## Abstract

The quality of polyacrylonitrile (PAN) precursor has a great influence on the properties of the resultant carbon fibers. In this paper, a novel comonomer containing the sulfonic group, 2-acrtlamido-2-methylpropane acid (AMPS), was introduced to prepare P(AN-co-AMPS) copolymers using itaconic acid (IA) as the control. The nanofibers of PAN, P(AN-co-IA), and P(AN-co-AMPS) were prepared using the electrospinning method. The effect of AMPS comonomer on the carbon nanofibers was studied using scanning electron microscopy (SEM), differential scanning calorimetry (DSC), Fourier transform infrared spectroscopy (FTIR), X-ray diffraction (XRD), thermogravimetric analysis (TGA) and Raman spectrum. The structural evolutions of PAN-based nanofibers were quantitatively tracked by FTIR and XRD during the thermal oxidative stabilization (TOS) process. The results suggested that P(AN-co-AMPS) nanofibers had the lower heat release rate (Δ*H/*Δ*T* = 26.9 J g^−1^ °C^−1^), the less activation energy of cyclization (*E_a_*_1_ = 26.6 kcal/mol and *E_a_*_2_ = 27.5 kcal/mol), and the higher extent of stabilization (*E_s_* and *SI*) during TOS process, which demonstrated that the AMPS comonomer improved the efficiency of the TOS process. The P(AN-co-AMPS) nanofibers had the better thermal stable structures. Moreover, the carbon nanofibers derived from P(AN-co-AMPS) precursor nanofibers had the better graphite-like structures (*X_G_* = 46.889). Therefore, the AMPS is a promising candidate comonomer to produce high performance carbon fibers.

## 1. Introduction

Due to their high strength and modulus, low weight, and excellent heat resistance, carbon fibers have been extensively used as a versatile reinforcing material of composites applied in the fields of aerospace, automotive industries, and civil engineering [1,2,3,4]. Polyacrylonitrile (PAN)-based precursor is one of the most important carbon fiber precursors, and PAN-based carbon fiber takes up almost 90% of the world’s high-performance carbon fiber market [5,6,7]. During the manufacturing process of PAN-based carbon fibers, thermal oxidative stabilization (TOS) at 200–300 °C is a key stage undergoing cyclization, dehydrogenation, oxidation, and tautomerization reaction [8,9]. The PAN macromolecules are converted from a linear structure to a non-meltable ladder-like structure which allows further carbonization at elevated temperature (1000–1700 °C) in an inert atmosphere [10,11]. However, PAN homopolymer undergoes an uncontrollable exothermic reaction because of the radical cyclization mechanism during the TOS process, which results in macromolecular chain scission, voids and defects in the final carbon fiber [12,13]. It is unfavorable not only for the high performance of carbon fiber but also for the manufacturing costs [14].

In order to solve this problem, several acidic comonomers, such as itaconic acid (IA) [15], acrylic acid (AA) [16], crotonic acid (CA) [17], 2-ethyl hexyl acrylate (EHA) [18], 2-methylenesuccinamic acid (MLA) [19], and vinyl acetate (VA) [20], have ever been incorporated into PAN macromolecular chains to improve the TOS using an ionic cyclization mechanism. Among these comonomers, IA with two carboxylic acid groups is the most efficient comonomer to initiate the cyclization of the nitrile group during the TOS process [21,22]. However, the scope of the study is mainly limited to the comonomers containing carbonyl functional units. Other acidic groups have been rarely reported. In our previous work [23], we found that ethylenesulfonic acid (ESA) containing a sulfonic group is more effective for PAN-based carbon fiber precursor than IA in the theoretical calculation and experimental results, including activation energy of cyclization, heat release, and char yielding. However, the cost of ESA limits its industrial application.

In the present work, we have further introduced another industrial-grade, low-cost comonomer into PAN copolymer used as a carbon fiber precursor, namely, 2-acrtlamido-2-methylpropane acid (AMPS) also containing the sulfonic group. AMPS is a hydrophilic vinyl monomer which is widely used in hydrogel [24]. To the best of our knowledge, the comonomer of AMPS has not been reported using for the fabrication of carbon fiber so far. Electrospinning is a simple and efficient technique for the fabrication of micro- to nano- scale fibers [25]. In the present study, the electrospinning method has been adopted to estimate the feasibility of the AMPS comonomer using for carbon fiber fabrication. Firstly, we prepared PAN, P(AN-co-IA), and P(AN-co-AMPS) (co)polymers and nanofibers by free-radical solution polymerization and electrospinning method, respectively. The morphologies of original nanofibers, pro-oxidation nanofibers, and carbonization nanofibers were studied by scanning electron microscopy (SEM). Next, the thermal stabilization mechanism and carbonization processes of these nanofibers were also studied in detail.

## 2. Materials and Methods

### 2.1. Materials

Itaconic acid (IA, analytical grade), 2,2-azobisisobutyronitrile (AIBN, analytical grade), and analytical grade methanol were obtained from Shanghai Macklin Biochemical Co., Ltd., Shanghai, China. Analytical grade acrylonitrile (AN), AMPS and dimethyl formamide (DMF, analytical grade) were purchased from Sinopharm Chemical Reagent Beijing Co., Ltd., Beijing, China. Dimethyl sulfoxide (DMSO, analytical grade) was purchased from Shanghai Aladdin Bio-Chem Technology Co., Ltd., Shanghai, China. AN was distilled under reduced pressure and restored in a freezer prior to further use. Other reagents were used without further purification.

### 2.2. Preparation of PAN Homopolymer and PAN Copolymers

**Scheme 1:**
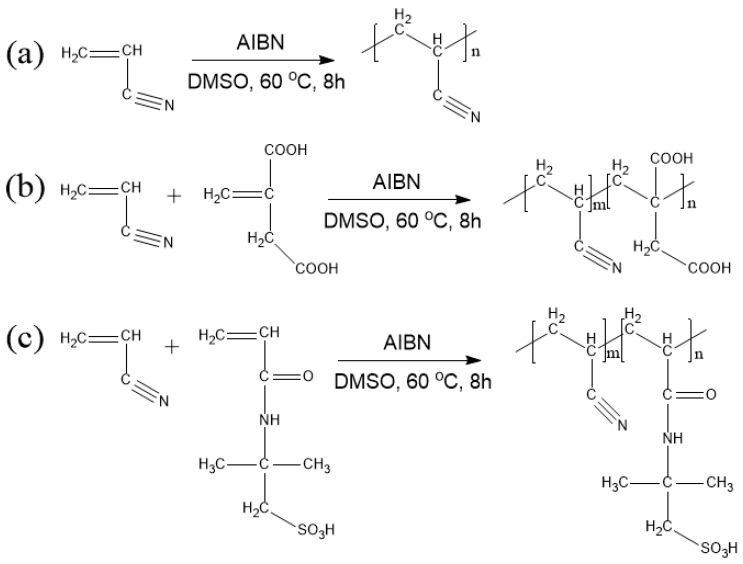
Synthesis of (**a**) polyacrylonitrile (PAN) homopolymer, (**b**) P(AN-co- itaconic acid (IA)), and (**c**) P(AN-co-2-acrtlamido-2-methylpropane acid (AMPS)) copolymers.

PAN homopolymer and PAN copolymers were synthesized by free-radical solution polymerization in a 100 mL three-necked flask. Briefly, by taking DMSO as a solvent and AIBN as an initiator (1 wt.% based on the monomers), the solvent concentration was 77 wt.%, and the molar ratio of AN and IA was 98:2. Next, the reaction was carried out at 60 °C at nitrogen atmosphere for 8 h with mechanical agitation. After polymerization, the substance produced was washed by deionized water and methanol to remove unreacted monomer, and then the substance was dried in a vacuum oven for 8 h. PAN homopolymer and P(AN-AMPS) were synthesized by the same method. The synthesis process of PAN homopolymer, P(AN-co-IA), and P(AN-co-AMPS) are shown in Scheme 1.

### 2.3. Preparation of PAN Homopolymer and PAN Copolymers Nanofibers

The electrospinning was performed using an electrospinning machine (TL-Pro, Shenzhen KONE Micro Technology Co., Ltd., Shenzhen, China). The electrospinning solution was prepared by dissolving PAN (co)polymers into DMF solvent and its concentration was 10 wt.%. The solution was loaded into a horizontal syringe with a 21-G metal nozzle. The applied voltage was 12 kV and the distance from the collector to the needle was 12 cm. The feed rate was 1 mL/h and the rotation rate of the roller was 1000 rpm. The resultant nanofibers were deposited onto the aluminum foil covering the roller. After electrospinning, as-obtained nanofibers were put into a vacuum oven at 60 °C for 12 h to remove the residual solvent.

### 2.4. Thermal Oxidative Stabilization and Carbonization

**Figure 1:**
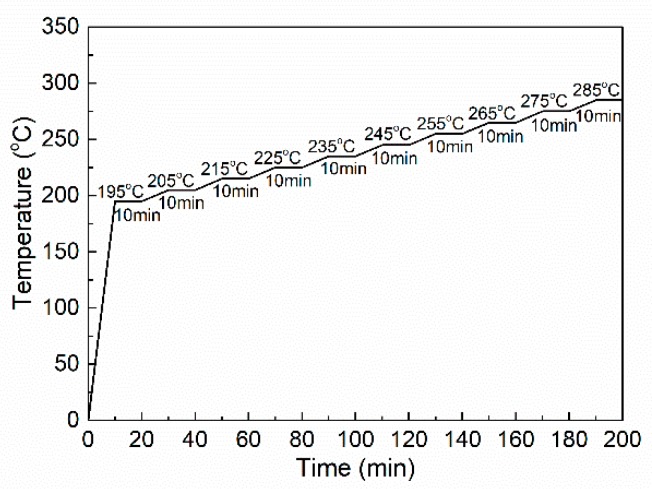
Heat treatment program for thermal stabilization of PAN and PAN copolymer nanofibers.

In order to study structural changes of PAN and PAN copolymer nanofibers during the thermal oxidative stabilization (TOS) process, all of the nanofibers were heated by the following program, as shown in Figure 1. The nanofibers after the TOS process are marked as oPAN, oP(AN-co-IA), and oP(AN-co-AMPS), respectively.

The carbonization of nanofibers was operated in a quartz tube oven at 1000 °C under a nitrogen atmosphere after TOS treatment of PAN and PAN copolymers nanofibers at 300 °C with 1 °C/min for 2 h in air condition. The nanofibers after carbonization treatment are marked as cPAN, cP(AN-co-IA), and cP(AN-co-AMPS), respectively.

**Scheme 2:**
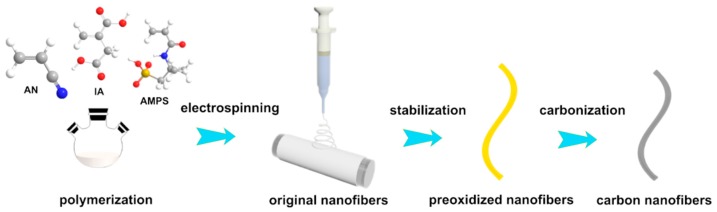
Schematic illustration of the processes of polymerization, electrospinning, stabilization, and carbonization.

The processes of the polymerization, electrospinning, stabilization, and carbonization are shown in Scheme 2.

### 2.5. Characterization

Gel permeation chromatography (GPC, Waters Breeze System, Mildford, MA, USA) was used to determine the molecular weight and molecular weight distribution of PAN homopolymer and PAN copolymers equipped with a refractive index detector using *N*,*N*′-Dimethylformamide as an eluent.

Field emission scanning electron microscopy (FE-SEM, JEOL 7500F, Tokyo, Japan) was used to study the morphology of all the nanofibers. Before SEM observation, all of the nanofibers were sputter-coated with platinum. The diameter and distribution of each nanofiber (at least 50 measurements) were measured using the SEM software SmileView (v2.5, JEOL, Tokyo, Japan).

Differential scanning calorimetry (DSC, DSC3, Mettler Toledo, Zurich, Switzerland) was used to study the thermal reaction during stabilization process ramped at 10 °C/min to 400 °C under nitrogen and air atmospheres, respectively.

The cyclization energy evaluation was performed using DSC (200 F3, Netzsch, Selb, Germany) from 25 °C to 400 °C under a nitrogen atmosphere at the heating rates of 5, 10, 15, 20 °C/min, respectively.

Fourier transform infrared spectrum (FTIR, Spectrum One Version B, PerkinElmer, Waltham, MA, USA) with attenuated total reflection (ATR) mode was recorded with a resolution of 4 cm^−1^ with 32 scans from 4000 to 500 cm^−1^ at different temperatures in the thermal stabilization process with the program (Figure 1).

X-ray diffraction (XRD, Rigaku MiniFlex600, Tokyo, Japan) was used to characterize the thermal stabilization process of PAN and PAN copolymers nanofibers with different temperatures, using the program (Figure 1). All the experiments were operated at a voltage of 40 kV and a current of 30 mA using nickel-filtered Cu-Kα radiation between 5 to 40° with a rate of 5 °C/min.

Thermogravimetric analysis (TGA, TGA2, Mettler Toledo, Zurich, Switzerland) was used to measure the weight loss of the nanofiber samples under nitrogen atmosphere ramped at 10 °C/min to 600 °C. 

The carbon structure of PAN-based carbon nanofiber carbonized at 1000 °C for 2 h was recorded by Raman spectrum (Renishaw Invia Plus laser Raman spectrometer, Renishaw, Wotton-under-Edge, UK) using 633-nm laser excitation. The peak fitting was operated using Origin Pro 9.0 software with Gaussian-LorenCross equation.

## 3. Results and Discussion

### 3.1. The Preparation of PAN-Based Nanofibers

The weight-average molecular weights (M_w_) of PAN homopolymer, P(AN-co-IA), and P(AN-co-AMPS) were 60,000 g/mol, 38,000 g/mol, and 31,000 g/mol, respectively, and their polydispersity index (PDI) values were 3.00, 2.92, and 2.58, respectively.

The incorporation of IA and AMPS comonomers into the PAN macromolecular chains reduces the values of M_w_, which can be attributed to the inhibition effect caused by the larger molecular volume of the comonomers. The solubilities of PAN homopolymer, P(AN-co-IA), and P(AN-co-AMPS) in DMF solvent are all good. The spinnability of the three polymers was evaluated by electrospinning as follows.

**Figure 2:**
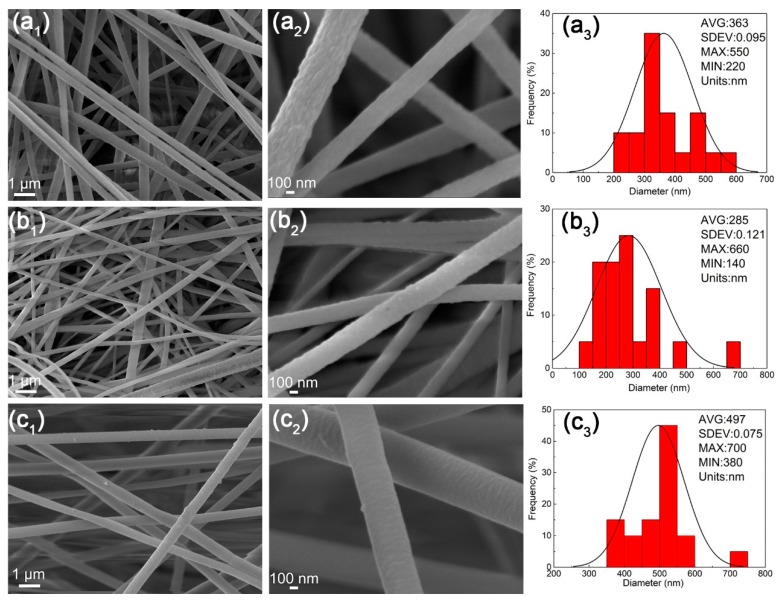
Representative scanning electron microscopy (SEM) images of (**a_1_**–**a_2_**) PAN, (**b_1_**–**b_2_**) P(AN-co-IA), and (**c_1_**–**c_2_**) P(AN-co-AMPS) nanofibers with different scales. (**a_3_**), (**b_3_**), and (**c_3_**) are diameter distribution histograms corresponding to PAN, P(AN-co-IA), and P(AN-co-AMPS) nanofibers, respectively.

Figure 2 shows the morphologies of the original PAN, P(AN-co-IA), and P(AN-co-AMPS) nanofibers. The diameters of PAN, P(AN-co-IA), and P(AN-co-AMPS) nanofibers were 363 ± 0.095 nm, 285 ± 0.121 nm, and 497 ± 0.075 nm, respectively. All of the PAN polymers were successfully electrospun to nanofibers with smooth surface and uniform diameter. This especially demonstrates the good spinnability of the novel P(AN-co-AMPS) copolymer. The fact that nanofibers made from different precursors have various diameters can be attributed to the different weight-average molecular weights (M_w_) and its distribution, electroconductibility of spinning dope, the viscoelastic force of the polymer solution, and the electrospinning conditions.

### 3.2. Thermal Stabilization Studies by DSC

**Figure 3:**
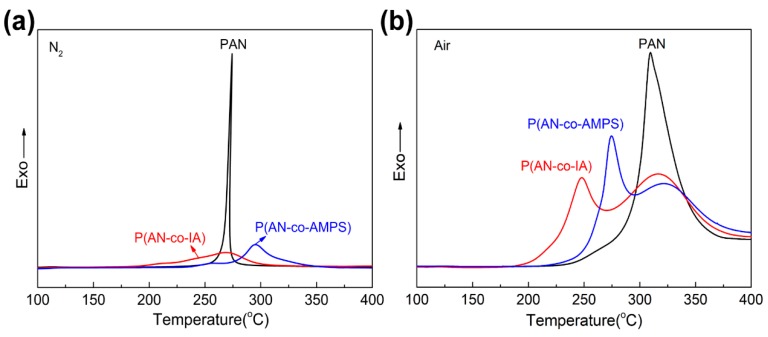
Differential scanning calorimetry (DSC) curves of PAN, P(AN-co-IA), and P(AN-co-AMPS) nanofibers under (**a**) N_2_ atmosphere and (**b**) air atmosphere.

Figure 3 shows the DSC thermograms of PAN, P(AN-co-IA), and P(AN-co-AMPS) nanofibers under N_2_ and air atmosphere, respectively. The corresponding parameters were obtained from the thermograms, including the temperature of initiation (*T_i_*), the temperature of termination (*T_f_*), and their difference (Δ*T*), as well as the peak temperature (*T_p_*), the released heat (Δ*H*), and the rate of heat release (Δ*H/ΔT*). The details are listed in Table 1.

**Scheme 3:**
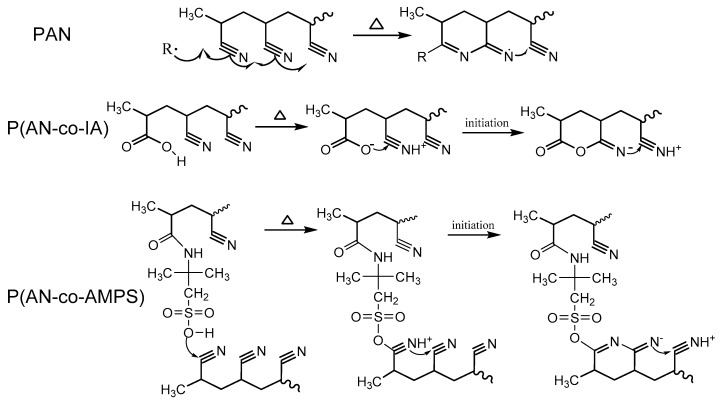
Cyclization reaction of PAN, P(AN-co-IA), and P(AN-co-AMPS) nanofibers through a free radical and an ionic cyclization mechanism.

Figure 3a shows the thermograms measurement under nitrogen atmosphere without the presence of oxygen, and principally includes cyclization and dehydrogenation reactions during this process [26]. As presented in Figure 3a, the single and narrow exothermic peak of PAN nanofibers can be attributed to the cyclization reaction initiated by the free radical mechanism (Scheme 3) [27]. The wide and multiple exothermic peaks of P(AN-co-IA) and P(AN-co-AMPS) nanofibers resulted from the cyclization reaction initiated by both the ionic and free radical mechanisms (Scheme 3) [28]. As shown in Table 1, the *T_i_* of P(AN-co-IA) and P(AN-co-AMPS) nanofibers was much lower than that of PAN nanofibers, which suggests an easier initiation of cyclization reaction in the novel P(AN-co-AMPS) nanofibers. Moreover, the Δ*T* and Δ*H* of PAN copolymers nanofibers were both larger than those of PAN homopolymer nanofibers. This means that there was a lower heat release rate (Δ*H/*Δ*T*) for PAN copolymers nanofibers during the cyclization process.

In the actual industrial production process, PAN undergoes the thermal oxidative stabilization (TOS) process under an air atmosphere, which principally includes cyclization, oxidation, and dehydrogenation reactions [29]. Figure 3b shows the DSC thermograms during the TOS process. It can be seen that all the peaks wholly shifted to higher temperatures and broadened in the presence of oxygen, implying a more violent exothermic process. The PAN nanofibers showed only one peak, while the P(AN-co-IA) and P(AN-co-AMPS) nanofibers presented wide and multiple peaks. The values of *T_i_* exhibited the same tendency as the results under nitrogen atmosphere (Table 1). In addition, the Δ*H/*Δ*T* values of P(AN-co-IA) and P(AN-co-AMPS) nanofibers were about 28.7 and 26.9 J g^−1^ °C^−1^, respectively, which are both lower than that of PAN nanofibers (103.7 J g^−1^ °C^−1^). It is noteworthy that the P(AN-co-AMPS) nanofibers had the lowest Δ*H* values, which is essential to avoid concentrative heat eruption and obtain a homogeneous stabilized structure. Therefore, the novel comonomer of AMPS can lower the cyclization temperature and retard the heat release during the TOS process, which is beneficial to produce high performance carbon fiber.

### 3.3. Evaluation of Activation Energy of Cyclization for PAN and PAN Copolymers Nanofibers

**Figure 4:**
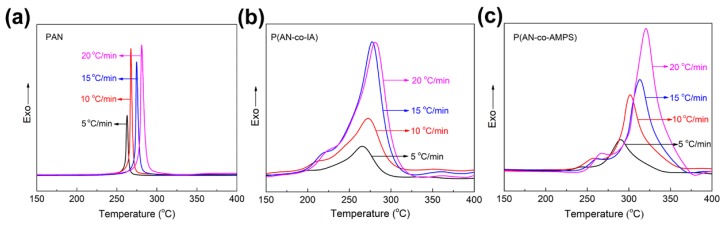
DSC curves of (**a**) PAN, (**b**) P(AN-co-IA), and (**c**) P(AN-co-AMPS) nanofibers heated at different rates under N_2_ atmosphere.

The apparent activation energy of cyclization reaction (*E_a_*) is an important parameter to evaluate the thermal behaviors of the PAN, P(AN-co-IA), and P(AN-co-AMPS) nanofibers [30,31]. In order to calculate the value of *E_a_*, the DSC curves with different heating rates (5, 10, 15 and 20 °C/min) under N_2_ atmosphere were collected, and are shown in Figure 4. It can be seen that the exotherms wholly moved to a higher temperature and the exothermic peaks became widen with the increase in heating rates.

The values of *E_a_* were calculated from the Kissinger equation (Equation (1) and the Ozawa equation (Equation (2) as follows [30,31]:

(1) $$-\frac{E_{a}}{R}=\frac{d[\ln(\varphi /T^{2})]}{d(1/T)}$$

**Figure 5:**
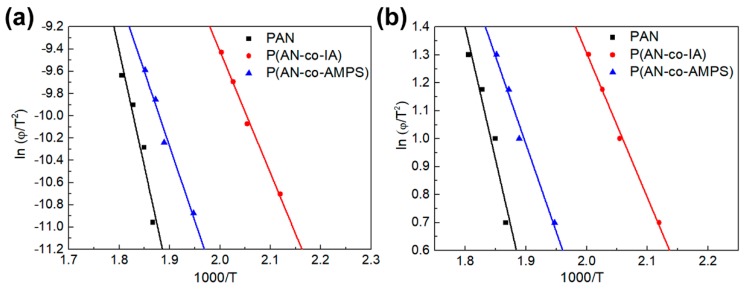
(**a**) Kissinger method and (**b**) Ozawa method for PAN, P(AN-co-IA), and P(AN-co-AMPS) nanofibers (plotting with Peak1).

*E_a_* was calculated from the slope of the linear plot of the $\ln(\varphi /T^{2})$ versus 1000/T, as shown in Figure 5a.

(2) $$-\frac{E_{a}}{R}=2.15\frac{d(\log\varphi )}{d(1/T)}$$

*E_a_* was calculated from the slope of the linear plot of log φ versus 1000/T as shown in Figure 5b. As in Equations (1) and (2), *R* is the gas constant, *T* is the maximum exothermic peak (absolute temperature), and φ is the heating rate.

The *E_a_* values of PAN, P(AN-co-IA), and P(AN-co-AMPS) nanofibers obtained using the Kissinger and Ozawa methods are listed in Table 2. As shown, the results from the Kissinger method and the Ozawa method are almost consistent. The *E_a_*_1_ obtained from the Kissinger method (Peak1) of PAN, P(AN-co-IA), and P(AN-co-AMPS) nanofibers were calculated as 41.2, 21.7, and 26.6 kcal/mol, respectively. The results of PAN and P(AN-co-IA) nanofibers almost agree with the values reported previously (40.2 kcal/mol for PAN [26] and 22.9 kcal/mol for P(AN-co-IA) [32]), respectively. The *E_a_*_1_ values of P(AN-co-IA) and P(AN-co-AMPS) nanofibers were both lower than that of PAN nanofibers. This indicates that the incorporation of AMPS comonomer improves the cyclization reaction the same as the IA comonomer. Moreover, compared with the *E_a_*_2_ values obtained from the second exothermic peak (Peak2), the *E_a_*_2_ values of P(AN-co-AMPS) nanofibers (27.5 kcal/mol) were lower than that of P(AN-co-IA) nanofibers (46.9 kcal/mol). This demonstrates that, as a whole, the cyclization reaction of PAN is more easily initiated by the AMPS comonomer containing the sulfonic group.

### 3.4. Structural Evolution during TOS Process by FTIR

**Figure 6:**
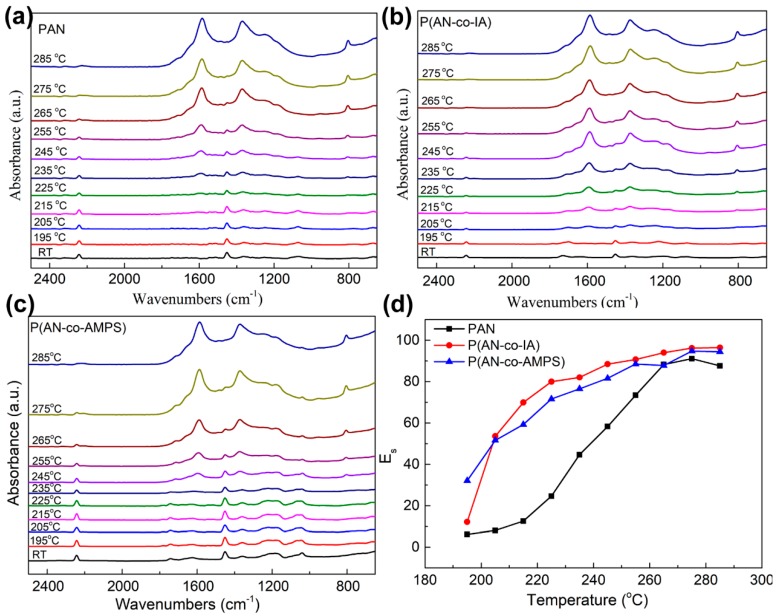
Fourier transform infrared spectroscopy (FTIR) spectra of (**a**) PAN, (**b**) P(AN-co-IA), and (**c**) P(AN-co-AMPS) nanofibers stabilized at different temperatures. (**d**) *E_s_* of PAN, P(AN-co-IA), and P(AN-co-AMPS) nanofibers at different temperatures.

Figure 6a–c show the FTIR spectra of PAN, P(AN-co-IA), and P(AN-co-AMPS) nanofibers stabilized at different temperatures for consecutive 10 min. As shown in the three original nanofibers spectra at room temperature (RT), the characteristic peak at 2243 cm^−1^ is attributed to the stretching vibration of nitrile group (C≡N) [33,34]. The characteristic peak at 1454 cm^−l^ was assigned to the symmetrical bending vibration of C-H in a hydrocarbon backbone [11]. These are the basic molecular structures of PAN. In the spectra of P(AN-co-IA), the characteristic peak at 1736 cm^−1^ was assigned to carboxyl (C=O) stretching vibration of the COOH groups [15]. The characteristic peak at 1034 cm^−1^ in the spectra of P(AN-co-AMPS) was due to S=O stretching vibration [35]. The above analyses indicate the presence of IA and AMPS comonomers in the target PAN macromolecules structure. 

As the stabilization temperature and time increased, the peak of stretching vibration of nitrile group gradually weakened and ultimately disappeared for all the PAN and PAN copolymer nanofibers. A new multiple and broad peak around 1590 cm^−1^ was generated, which was assigned to the stretching vibration of C=C conjugated with those of C=N and N-H groups originating from cyclization, dehydrogenization, and tautomerization reactions [11]. The weakened peak intensity of C-H at 1454 cm^−1^ originated from the dehydrogenation reaction [36]. The appearance of another obvious wide peak around 1370 cm^−1^, assigned to C-H, N-H, and O-H in the ring, also demonstrated the occurrence of the cyclization reaction [37]. A weak peak at 810 cm^−1^ gradually appeared with thermal treatment, which was assigned to the out-of-plane bending of =C-H in saturated rings [11]. All the findings clearly indicate that cyclization, oxygen uptake or dehydrogenation occurred during the TOS process.

In order to quantitatively study the structural evolution of PAN and PAN copolymer nanofibers during the TOS process, the extent of stabilization (*E_s_*) was calculated using the following equation [38]:

(3) $$E_{s}=\frac{f*A_{1590}}{A_{2243}+f*A_{1590}}$$

where *A*_1590_ = absorbance intensity of C = C + C = N + N-H, *A*_2243_ = absorbance intensity of C≡N, and *f* is the ration of C≡N and C = C + C=N + N-H group absorptivity constants = 0.29.

The plot of *E_s_* of PAN, P(AN-co-IA), and P(AN-co-AMPS) nanofibers at different temperatures during the TOS process is shown in Figure 6d. Generally, as the stabilization temperature increased, the values of *E_s_* gradually increased for all of the nanofibers, suggesting the transformation from C≡N structure to C=C, N-H, and C=N structures. What’s more, the *E_s_* values of P(AN-co-IA) and P(AN-co-AMPS) nanofibers were higher than that of PAN in all of the temperature ranges. This demonstrates that the cyclization reaction of the C≡N group is more easily initiated by the incorporation of IA and AMPS comonomers. The *E_s_* value of P(AN-co-AMPS) nanofibers (32.1) was higher than that of P(AN-co-IA) nanofibers (12.2) at 195 °C. This indicates that the AMPS comonomer immediately triggers the cyclization reaction after heating. The TOS efficiency of P(AN-co-AMPS) nanofibers was higher than that of P(AN-co-IA) nanofibers. Those with higher *E_s_* value and less unreacted C≡N group avoided thermolysis and the forming of defects in the stage of carbonization [36].

### 3.5. Structural Evolution during TOS Process by XRD

**Figure 7:**
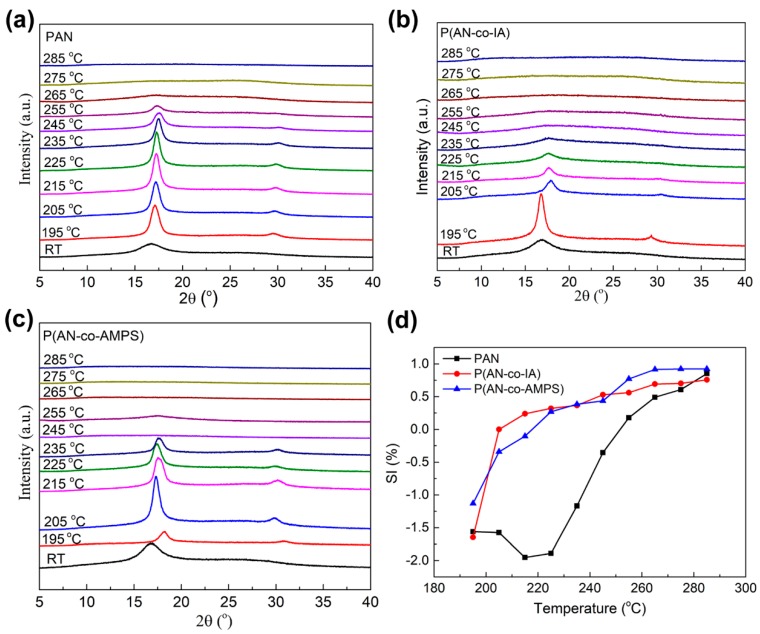
XRD patterns of (**a**) PAN, (**b**) P(AN-co-IA), and (**c**) P(AN-co-AMPS) nanofibers stabilized at different temperatures. (**d**) *SI* of PAN, P(AN-co-IA), and P(AN-co-AMPS) nanofibers at different temperatures.

Figure 7a–c display the XRD patterns of PAN, P(AN-co-IA), and P(AN-co-AMPS) nanofibers stabilized at different temperature for consecutive 10 min. At RT, the XRD patterns of the three original nanofibers showed a broad diffraction peak at about 2θ = 17° which was attributed to the (100) plane of the pseudo-hexagonal lattice of the C≡N groups [39,40]. As the stabilization temperature and time increased, the peak parameters, including peak intensity, peak position, and the number of peaks, underwent obvious. In particular, the new minor peaks appeared at approximately 2θ = 30° after the nanofibers were heat treated at 195 °C for 10 min, which corresponds to the production of (110) crystalline plane of the pseudo-hexagonal cell [41]. In the temperature range of 195 °C to 255 °C (235 °C for P(AN-co-IA) and P(AN-co-AMPS) nanofibers), the (100) peak shifted to a higher Bragg angle indicating a smaller interlayer spacing (*d*). This indicates that the polymer chain segments rearranged at an elevated temperature during the TOS process.

As mentioned above, the TOS process converts the linear structure to a ladder structure, which can be related to the changes of peak intensity of (100) plane [42]. Equation (4) is used to evaluate the extent of stabilization during the TOS process, as follows [41]:

(4) $$SI=\left(I_{0}-I_{s}\right)/I_{0}$$

where *I*_0_ is the peak intensity of (100) plane from the original nanofibers and *I_s_* is the peak intensity of (100) plane from the nanofibers stabilized at different temperatures. Figure 7d shows the *SI* of PAN, P(AN-co-IA), and P(AN-co-AMPS) nanofibers as a function of temperatures during the TOS process. As shown in Figure 7d, all of the *SI* of PAN, P(AN-co-IA), and P(AN-co-AMPS) nanofibers at 195 °C are negative. This might be because the energy supplied by heating is not enough to start the TOS process, but is enough to activate chain segment motion and further improve the recrystallization [19]. As the temperature increased, the *SI* of P(AN-co-IA) and P(AN-co-AMPS) nanofibers gradually increased. It means the thermal stabilization reaction accompanied by the transition from the linear structure to ladder structure. However, the *SI* of PAN decreased below 235 °C, which indicates that the stabilization reaction is more difficult than the recrystallization. It also demonstrates the lower initiation stabilization temperature and the higher TOS efficiency of P(AN-co-IA) and P(AN-co-AMPS) nanofibers than those of PAN. The results agree with the analyses using FTIR.

### 3.6. Thermal Behaviors Studies by TGA

**Figure 8:**
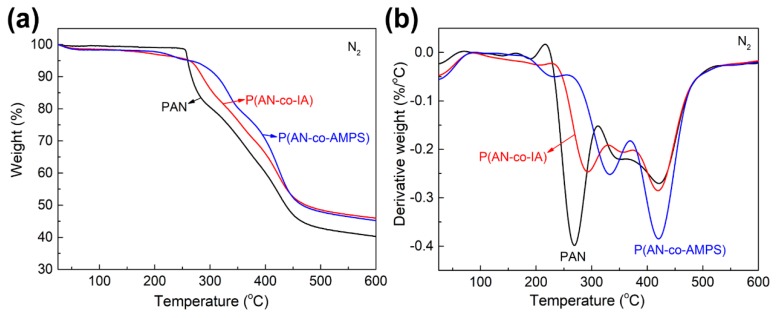
Thermogravimetric analysis (TGA) (**a**) and derivative thermogravimetric (DTG) (**b**) curves of PAN, P(AN-co-IA), and P(AN-co-AMPS) nanofibers under the N_2_ atmosphere, respectively.

Figure 8 shows the thermal behaviors of PAN, P(AN-co-IA), and P(AN-co-AMPS) nanofibers under N_2_ atmosphere. The slight weight loss at temperature under 100 °C of P(AN-co-IA) and P(AN-co-AMPS) nanofibers is owing to the moisture evaporation derived from the hydrophily of IA and AMPS comonomers. Due to the absence of oxygen molecule, the weight loss at this heat treatment process can be attributed to the dehydrogenation and thermal decomposition [43]. The temperature of initial weight loss for P(AN-co-IA) and P(AN-co-AMPS) nanofibers are lower than that for PAN nanofibers. This can be attributed to the comonomers of IA and AMPS promoting the dehydrogenation reaction to a certain extent [14]. As shown in Figure 8b, the temperatures of the first maximum weight loss rate (*T_w_*) of PAN, P(AN-co-IA), and P(AN-co-AMPS) nanofibers were about 260.8 °C, 280.5 °C, and 335.3 °C, respectively. The lowest *T_w_* of PAN nanofibers could be due to the violent free radical cyclization mechanism, which quickly releases plenty of heat and leads to thermal degradation of the PAN chains [44]. In contrast, the P(AN-co-AMPS) nanofibers have the highest *T_w_* suggesting the best thermal stable structures. The second *T_w_* of the three nanofibers are around 420 °C, which is due to the degradation and produces lots of volatile particles. In the end, the final weight residual at 600 °C of PAN, P(AN-co-IA), and P(AN-co-AMPS) nanofibers were about 40.3 wt.%, 45.9 wt.%, and 45.2 wt.%, respectively. This indicates that the addition of AMPS comonomer improved the cyclization of PAN under the same heating conditions, generating more heat-resistant molecular structures and favoring further carbonization.

### 3.7. The Morphologies of Nanofibers after TOS Process

**Figure 9:**
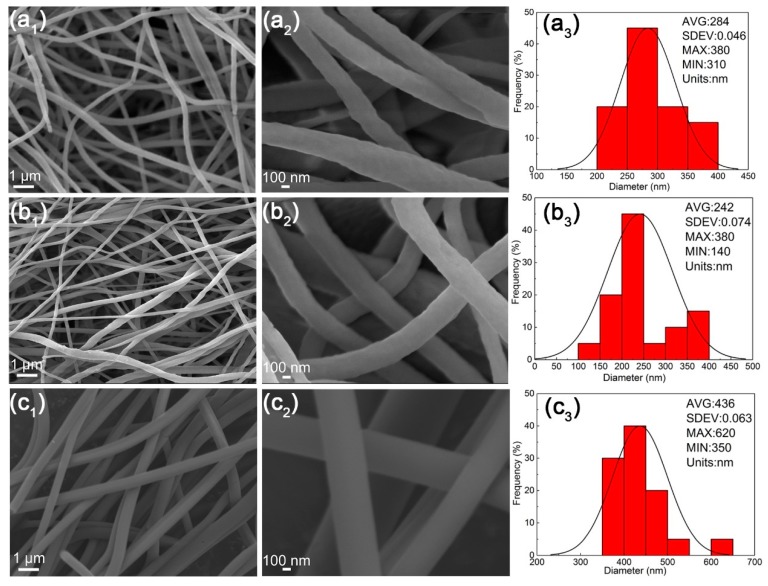
Representative SEM images of (**a1**–**a2**) oPAN, (**b1**–**b2**) oP(AN-co-IA), and (**c1**–**c2**) oP(AN-co-AMPS) nanofibers with different scales. (**a3**), (**b3**), and (**c3**) are diameter distribution histograms corresponding to oPAN, oP(AN-co-IA), and oP(AN-co-AMPS) nanofibers, respectively.

Figure 9 shows the morphologies of nanofibers after the TOS process. The diameters of oPAN, oP(AN-co-IA), and oP(AN-co-AMPS) nanofibers are 284 ± 0.046 nm, 242 ± 0.074 nm, and 436 ± 0.063 nm, respectively. Compared to the original nanofibers, the diameters of pro-oxidized nanofibers all gradually decrease, which could be due to the dehydrogenation and the structure evolution.

### 3.8. The Morphologies of Nanofibers after Carbonization

**Figure 10:**
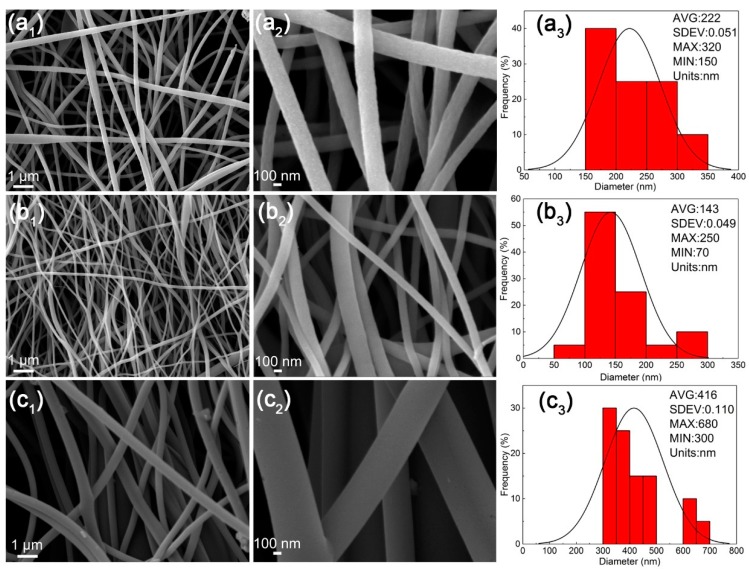
Representative SEM images of (**a_1_**–**a_2_**) cPAN, (**b_1_**–**b_2_**) cP(AN-co-IA), and (**c_1_**–**c_2_**) cP(AN-co-AMPS) nanofibers with different scales. (**a_3_**), (**b_3_**), and (**c_3_**) are diameter distribution histograms corresponding to cPAN, cP(AN-co-IA), and cP(AN-co-AMPS) nanofibers, respectively.

Figure 10 shows the morphologies of nanofibers after carbonization treatment. It is known that the TOS process causes the cyclization of nitrile groups and forms the thermally stable ladder-like structure which avoids fusing during the carbonization process [45,46]. The diameters of cPAN, cP(AN-co-IA), and cP(AN-co-AMPS) nanofibers were 222 ± 0.051 nm, 143 ± 0.049 nm, and 416 ± 0.110 nm, respectively. The shrinkage of the nanofiber diameters could be due to the degradation and exclusion of non-carbon atoms [47].

### 3.9. The Carbon Structure of Nanofibers after Carbonization

**Figure 11:**
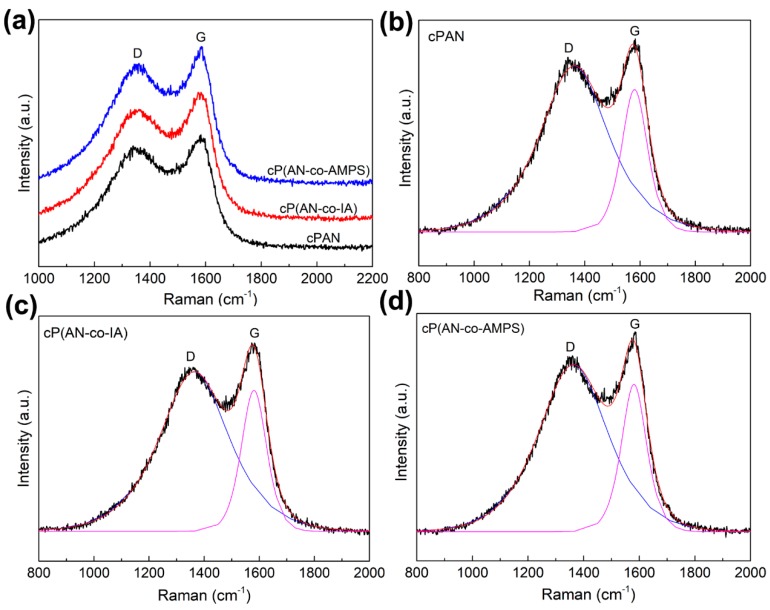
The Raman spectrum (**a**) and the corresponding fitting peaks of (**b**) cPAN, (**c**) cP(AN-co-IA), and (**d**) cP(AN-co-AMPS) nanofibers. (The blue and pink solid lines are the single fitting peaks; the red solid line is the superposition of the two fitting peaks.).

The Raman spectra of cPAN, cP(AN-co-IA), and cP(AN-co-AMPS) nanofibers carbonized at 1000 °C for 2 h are shown in Figure 11. The peak parameters obtained from the fitting Raman curves such as the intensity ratio of the D and G peaks (I_D_/I_G_), the full width at half maximum (FWHM) of the D and G peaks, the in-plane graphitic crystallite size (*L_a_*), and the mole fraction of graphite (*X_G_*) of the cPAN, cP(AN-co-IA), and cP(AN-co-AMPS) nanofibers are listed in Table 3. The peaks around 1350 cm^−1^ (D band) are associated with a disordered diamond-like carbon structure, while the peaks around 1580 cm^−1^ (G band) are attributed to an ordered carbon cluster. The I_D_/I_G_ value indicates the number of defects in the ordered carbon clusters of the carbon nanofibers [39,48]. The I_D_/I_G_ values of cPAN, cP(AN-co-IA), and cP(AN-co-AMPS) nanofibers were 1.166, 1.132, and 1.133, respectively. The higher the I_D_/I_G_ values, the more defects in the ordered carbon clusters [38,49]. The I_D_/I_G_ value of cPAN nanofibers was higher than those of cP(AN-co-IA) and cP(AN-co-AMPS) nanofibers. The *L_a_* value was inversely proportional to the I_D_/I_G_ value. The *X_G_* values of cP(AN-co-IA) and cP(AN-co-AMPS) nanofibers were larger than that of cPAN nanofibers. The content of graphitic sp^2^ carbon crystal can be estimated from the FWHM of the D and G peaks. The relatively sharper D and G peaks of cP(AN-co-IA) and cP(AN-co-AMPS) nanofibers suggest the higher degrees of crystallinity [39]. In essense, it indicated that the comonomers of IA and AMPS improve the thermal stabilization and produce better graphite-like structures after the carbonization process.

## 4. Conclusions

In this study, a novel PAN-based carbon fiber precursor of P(AN-co-AMPS) copolymer was prepared. Moreover, the thermal stabilization and carbonization processes of the electrospinning nanofibers were investigated. The SEM showed that all of the nanofibers had a smooth surface and uniform diameter in the nanoscale. The DSC test indicated that the P(AN-co-AMPS) nanofibers had the lowest released heat (Δ*H*) and a lower heat release rate (Δ*H/ΔT*), especially, under an air atmosphere. Based on the Kissinger and Ozawa methods, the results demonstrated that the P(AN-co-AMPS) nanofibers had less activation energy of cyclization (*E_a_*_1_ = 26.6 kcal/mol and *E_a_*_2_ = 27.5 kcal/mol), which indicated that the cyclization reaction of PAN was more easily initiated by the AMPS comonomer containing the sulfonic group. Furthermore, P(AN-co-AMPS) nanofibers had a higher extent of stabilization (*E_s_* and *SI*) during the TOS process, quantitatively tracked by FTIR and XRD. TGA results showed the P(AN-co-AMPS) nanofibers had better thermal stable structures. The Raman test indicated that the carbon nanofibers derived from P(AN-co-AMPS) precursor nanofibers had better graphite-like structures (*X_G_* = 46.889). The shrinkage of the nanofiber diameters after carbonization could be due to the degradation and exclusion of non-carbon atoms. Based on the above results, AMPS is a promising comonomer to improve the TOS process of PAN. Furthermore, the P(AN-co-AMPS) copolymer could be used for the fabrication of high-performance carbon fibers in the industry.

## Figures and Tables

**Table 1:** DSC results of PAN, P(AN-co-IA), and P(AN-co-AMPS) nanofibers under N_2_ and air atmospheres, respectively.

Polymer Codes		*T_i_* (°C)	*T_p_*_1_ (°C)	*T_p_*_2_ (°C)	*T_f_* (°C)	Δ*T* (°C)	Δ*H* (J/g)	Δ*H*/Δ*T* [J/(g °C)]
PAN	N_2_	268.4	274.3		279.5	11.1	534.1	48.1
P(AN-co-IA)		217.4	212.1	268.8	300.6	83.2	610.0	7.3
P(AN-co-AMPS)		240.5	257.6	295.2	318.9	78.4	641.0	8.2
PAN	Air	297.1	309.3		345.2	48.1	4988.8	103.7
P(AN-co-IA)		222.6	247.6	316.6	400.9	178.3	5110.7	28.7
P(AN-co-AMPS)		252.9	274.5	321.4	401.0	148.1	3987.5	26.9

**Table 2:** Activation energy calculated from Kissinger and Ozawa methods.

Polymer Codes	Kissinger (kcal/mol)		Ozawa (kcal/mol)	
	Peak1	Peak2	Peak1	Peak2
PAN	41.2		40.4	
P(AN-co-IA)	21.7	46.9	22.1	46.0
P(AN-co-AMPS)	26.6	27.5	26.8	27.8

**Table 3:** The detailed peak parameters of cPAN, cP(AN-co-IA), and cP(AN-co-AMPS) nanofibers obtained from the fitting Raman curves.

Codes	I_D_/I_G_	*L_a_*^a^ (nm)	*X_G_*^b^ (%)	D Peak FWHM (cm^−1^)	G Peak FWHM (cm^−1^)
cPAN	1.166	3.77	46.168	296.5	115.2
cP(AN-co-IA)	1.132	3.89	46.905	283.4	111.6
cP(AN-co-AMPS)	1.133	3.88	46.889	290.6	109.1

^a^*L*_a_ = 4.4 × (I_D_/I_G_)^−1^ [50]. ^b^ *X_G_* = (I_G_ × 100/(I_G_ + I_D_)) [50].
